# Fungal chitin is not an independent mediator of allergic fungal asthma severity

**DOI:** 10.1152/ajplung.00041.2024

**Published:** 2024-06-25

**Authors:** Diandra A. Ellis, MaryJane Jones, Hubertine M. E. Willems, Suki Cheung, Mgayya Makullah, Vishukumar Aimanianda, Chad Steele

**Affiliations:** ^1^Department of Microbiology and Immunology, School of Medicine, Tulane University, New Orleans, Louisiana, United States; ^2^Unité de Mycologie Moléculaire, Institut Pasteur, Université de Paris, CNRS, UMR2000, Paris, France

**Keywords:** allergy, asthma, fungi, immune

## Abstract

Chitin, a polysaccharide found in the fungal cell wall and the exoskeletons of house dust mites and cockroaches, has garnered attention as a potential immunoreactive allergen. Mammals have evolved to express chitin-degrading chitinases (acidic mammalian chitinase/AMCase and chitotriosidase) that may modulate immune responses to chitin. We have previously reported that mice deficient in AMCase (*Chia^−^*^/−^) demonstrated better lung function during allergic fungal asthma. As expected, we show that mice overexpressing AMCase (SPAM mice) had worse airway hyperreactivity (AHR) during allergic fungal asthma. We further demonstrate that chitin-positive *Aspergillus fumigatus* conidia are detectable in the allergic lung during chronic exposure. Lung function in *Chia^−^*^/−^ and SPAM mice is directly correlated with the level of chitinase activity during chronic fungal exposure (*Chia^−^*^/−^ mice, negligible chitinase activity, lower AHR; SPAM mice, heightened chitinase activity, higher AHR), suggesting that the breakdown of chitin promoted AHR. However, chronic exposure of normal mice to purified *A. fumigatus* chitin resulted in only moderate inflammatory changes in the lung that were not sufficient to induce AHR. Moreover, despite having dramatic differences in chitinase activity, chronic exposure of *Chia^−^*^/−^ and SPAM mice to purified *A. fumigatus* chitin likewise did not modulate AHR. Collectively, these results indicate that chronic exposure to fungal chitin alone is incapable of driving AHR. Furthermore, our data suggest that the chitinase-mediated degradation of chitin associated with *A. fumigatus* conidia may facilitate unmasking and/or liberation of other fungal cell wall components that drive inflammatory responses that contribute to AHR.

**NEW & NOTEWORTHY** Humans with asthma sensitized to fungi often have more severe asthma than those who are not fungal-sensitized. Chitin makes up a significant portion of the cell wall of fungi and has been implicated as a pathogenic factor in allergic asthma. Ellis et al. demonstrate that chronic exposure to fungal chitin alone is unable to modulate lung function, even in the presence of differential lung chitinase activity.

## Graphical abstract

**Figure:**
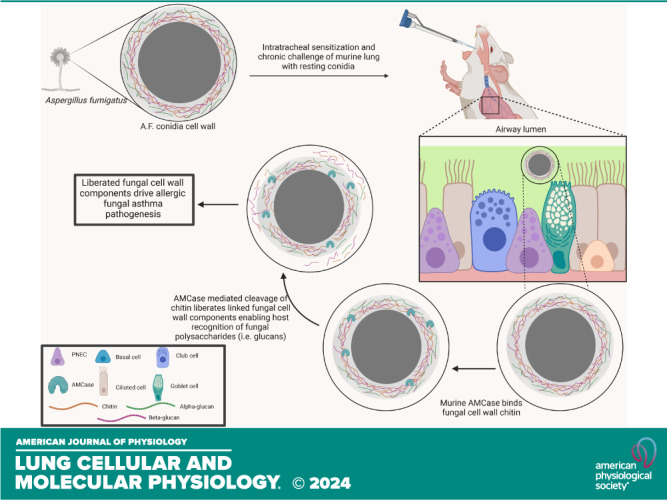

## INTRODUCTION

Asthma is a spectrum of chronic inflammatory diseases that is subcategorized into multiple endotypes and affects more than 300 million people worldwide (1). Allergic asthma is a large subset of asthmatics comprising nearly two-thirds of all cases and includes sensitization to a variety of trees, plants, pets, molds, and insects (2). Severe asthma with fungal sensitization (SAFS) defines a subset of allergic asthmatics with poorly controlled symptoms who are sensitized to any one or more fungal geni, such as *Aspergillus*, *Cladosporium*, or *Penicillium* (3, 4). Although *Aspergillus spp.* are the most prominent fungal allergens associated with fungal asthma, SAFS afflicts >6 million people worldwide (5, 6). We originally used an experimental animal model of allergic fungal asthma and reported that the fungal β-glucan receptor Dectin-1- and Dectin-1-mediated IL-22 drove the severity of allergic fungal asthma (7). Since this report, we have identified mediators such as IL-7 (8) and IL-1α/IL-1β (9) in fungal-sensitized human asthmatics that drove immunopathogenic responses during experimental allergic fungal asthma whereas mediators such as interleukin-1 receptor antagonist (IL-1RA) (9) and CX3CL1 (10) limited the severity of allergic fungal asthma.

Chitin is the second most abundant polysaccharide to cellulose, and is synthesized by diverse organisms such as fungi, parasites, crustaceans, and insects (11, 12). Although mammals lack the ability to synthesize chitin, expression of chitinases and chitinase-like-proteins (C/CLPs) have been conserved across evolution (13). The glycoside hydrolase 18 family (GH18) includes two chitinases, chitotriosidase (*Chit1*) and acidic mammalian chitinase (*Chia*, AMCase), that can sequester and enzymatically cleave chitin. Other GH18 family members include several CLPs, such as BRP-39 (*Chi3l1*) and Ym-1, which bind chitin and other complex lectins, but lack enzymatic activity. Multiple reports have demonstrated elevated levels of C/CLPs to be prognostic biomarkers of inflammatory disease and indicators of asthma severity (14–16). We have previously reported that deficiency in AMCase/*Chia* was associated with less severe allergic fungal asthma (17). Furthermore, we reported that deficiency in BRP-39/*Chi3l1* was associated with more severe allergic fungal asthma (18). Collectively, our data suggest that AMCase contributes to allergic fungal asthma severity whereas BRP-39 functions to minimize severity. However, the relationship between these observations and the role of chitin have yet to be determined.

Efforts to better understand the role of chitin in allergic asthma severity have used multiple preparations and sources of chitin, including shellfish-derived chitin or insect exoskeleton chitin extracts, beads coated with chitin and chitin fragments of varying sizes, varying exposure regimens, and administering chitin concomitant with an additional allergen (11, 19–23). Overall, data to date place limits on the interpretations of these reports as it relates to chronic exposure to aeroallergen-derived chitin. Fungal chitin administered acutely to the lung has been assessed previously (11), although the purity of the preparation was questionable. In the current report, we used a highly purified (β-glucanase treated) and highly acetylated (>90%) chitin preparation extracted from *Aspergillus fumigatus* hyphae (24), which we have previously reported to have higher chitin content than *A. fumigatus* conidia (17), to determine the effects of chronic fungal chitin administration on lung inflammatory and physiologic responses.

## MATERIALS AND METHODS

### Mice

Wild-type (WT) C57BL/6 control mice and AMCase-deficient (*Chia*^−/−^) mice were used as we have previously described (17, 18). SPAM mice (AMCase overexpressing) were a kind gift from Dr. Steve Van Dyken, Washington University (23, 25) and bred at Tulane. SPAM (Tg+/0) hemizygous mice were bred to wild-type C57BL/6 mice to yield SPAM (Tg+/0) and littermate control (0/0) mice. Primer sequences for genotyping SPAM mice were as follows: 5′AMCase-2, 5′-GAG CAG GAG GCT ATT GAG AG-3′ and 3′AMCase-BHI, 5'-CGG GAT CCG GTT CAT GGC CAG TTG-3′. SPAM (Tg+/0) mice were identified by the presence of the SPAM transgene (700 bp) on a 1% agarose gel. Male and female mice, 6–12 wk of age, were used for all experiments. Animal housing was in a specific-pathogen free facility under Association for Assessment and Accreditation Laboratory Animal Care certification. Animals were handled according to Public Health Service Office of Laboratory Animal Welfare policies after review and approval by the Tulane Institutional Animal Care and Use Committee.

### Preparation of *A. fumigatus* and Chronic In Vivo Exposure

*Aspergillus fumigatus* isolate 13073 (ATCC, Manassas, VA) was maintained on potato dextrose agar for 5–7 days at 37°C. Conidia were harvested by washing the culture flask with 50 mL of sterile phosphate buffered saline supplemented with 0.1% Tween 20. The conidia were then passed through a sterile 40-μm nylon membrane to remove hyphal fragments and enumerated on a hemacytometer. The repeated *A*. *fumigatus* associated allergic airway inflammation model was performed as we have extensively reported (7–10, 17, 18). Briefly, mice were lightly anesthetized with isoflurane and administered 1 × 10^7^ live *A. fumigatus* conidia in a volume of 50 μL of PBS intratracheally (it). After resting for 7 days, mice were challenged intratracheally with 1 × 10^6^ live *A. fumigatus* conidia in 50 μL of PBS daily for five consecutive days (days 7, 8, 9, 10, and 11), allowed to rest for two consecutive days (*days 12* and *13*), and then challenged intratracheally with 1 × 10^6^ live *A. fumigatus* conidia in 50 μL of PBS daily for three consecutive days (*days 14*, *15*, and *16*). Twenty-four hours after the last *A. fumigatus* challenge (*day 17*), mice were euthanized for assessment of various outcome measures.

### *Aspergillus fumigatus* Chitin Preparation

Chitin was isolated from *A. fumigatus* as previously described (24). Briefly, *A. fumigatus* strain CEA17_ΔakuBKU80 was cultured for 20 h at 37°C in liquid Sabouraud medium. Mycelia were washed three times with deionized water, resuspended in 5% (wt/vol) potassium hydroxide, and boiled for 30 min at 100°C. The preparation was then centrifuged at low speed, and the pellet was washed three times with deionized water, resuspended in 40% H_2_O_2_-glacial acetic acid (1:1) solution, and autoclaved for 20 min at 121°C. Material was collected by centrifugation, the pellet was washed with water, and the sample was extracted a further three times in hot 5% potassium hydroxide with washes between each boiling step. Finally, the chitin preparation was washed repeatedly, suspended in 0.5 mL of 50 mM acetate buffer, pH 6.0, and treated with endo-β-(1,3)-glucanase (20 µL containing 5 µg protein) at 37°C overnight and the absence of β-(1,3)-glucan was confirmed by gas chromatographic analysis. The degree of acetylation in the chitin preparation was determined to be >90% as previously described (24). Likewise, the sizes of the chitin particles were determined to be <0.1 µm (∼31%), >0.1 µm to <0.5 µm (∼43%), >0.5 µm to <3.0 µm (∼12%), >3.0 µm to <6.0 µm (∼7%), and >6.0 µm to <15.0 µm (∼7%). Chitin was reconstituted in sterile saline at 1 µg/µL and sonicated twice for 5 min in a Branson 200 Ultrasonic Cleaner. Mice were challenged intratracheally with 50 µg of chitin in the same regimen as *A. fumigatus* conidia.

### Pulmonary Function Assessment

Individual anesthetized *A*. *fumigatus-*exposed mice were intubated, and each animal was attached to a computer-controlled volume ventilator (flexiVent; SCIREQ). Regular breathing was set at 150 breaths/min, with volume and pressure controlled by the flexiVent system based on individual animal weights. Positive end-expiratory pressure was set to 2 cmH_2_O and measured during each breath stroke. The single-frequency forced oscillation technique was used to measure total/dynamic lung resistance (Rrs). The low-frequency/broadband forced oscillation technique was used to measure Newtonian resistance (Rn; also known as airway hyperreactivity, AHR). All measurements were collected at baseline and after a linear dose response with methacholine challenge (10–50 mg/mL), as previously described (7–10, 17, 18). Lung function was also assessed in naive WT and mutant mice, which confirmed no baseline anomalies and no differences between groups (data not shown).

### Bronchoalveolar Lavage for Lung Cell Flow Cytometry Staining

Lung cells were isolated via bronchoalveolar lavage (BAL) as previously described (18). Cells were washed, and Fc receptors were blocked with Mouse BD Fc Block (BD Biosciences) at 4°C for 20 min. Thereafter, cells were stained with a single-color LIVE/DEAD Fixable Dead Cell Stain (Invitrogen), followed by labeling with specific immune cell surface markers. Cells were identified as follows: neutrophils—CD45+, CD11b+, Siglec F−, Ly6G+; eosinophils—CD45+, CD11b+, Ly6G−, Siglec F+; dendritic cells—CD45+, CD11c+, Ly6C+, MHCII+; and CD4 T cells—CD45+, CD3+, CD4+, TCRb+ (antibodies from Biolegend, San Diego, CA and Miltenyi Biotec, Bergisch Gladbach, Germany). Samples were acquired using a 4-laser, 20-parameter analytic BD LSRFortessa, and data were analyzed using FlowJo software (Tree Star Inc., Ashland, OR). Unstained lung leukocytes served as a control for background fluorescence and gating. Appropriately stained UltraComp eBeads (Thermo Fisher Scientific, Waltham, MA) served as single-color controls.

### Lung Samples for Luminex Analysis

Cytokines were quantified in either lung digest cell cultures or lung homogenates. For lung cell isolation, the lungs were collected and minced in IMDM media (MilliporeSigma) supplemented with 1× penicillin-streptomycin-glutamine (Mediatech), 10% heat-inactivated FBS (Invitrogen), and 0.4 mg/mL polymyxin B (Thermo Fisher Scientific), followed by incubation for 60 min with tissue culture grade type IV collagenase (1 mg/mL; MilliporeSigma) in a 37°C orbital shaker at 100 rpm. The cell suspension was filtered through sterile 70-μm and 40-μm nylon filters, and red blood cells were lysed with ACK buffer (Lonza) to create single-cell preparations. One million cells in a volume of 200 μL were cultured overnight with 1 million *A. fumigatus* conidia (1:1), followed by collection and clarification of supernatants. For lung homogenates, the right lung was homogenized in PBS supplemented with Complete Mini protease inhibitor tablets (Roche Diagnostics), clarified by centrifugation (12,000 *g* for 10 min at 4°C), and stored at −80°C. Cell culture supernatants or clarified lung homogenate supernatants were analyzed for the protein levels of 32 cytokines and chemokines using the Luminex-based Milliplex multiplex suspension cytokine array (MilliporeSigma), according to the manufacturer’s instructions. The data were analyzed using Bio-Plex Manager software (Bio-Rad). IL-33, CCL17, and CCL22 levels were quantified by ELISA (R&D Systems).

### Bronchoalveolar Lavage for Chitinase Activity

BAL fluid (BALF) was collected using PBS supplemented with Complete Mini protease inhibitor tablets (Roche Diagnostics) and centrifuged at 12,000 *g* for 10 min at 4°C. Supernatants were collected and frozen at −80°C and subsequently shipped to Dr. Steve Van Dyken, Washington University, for analysis as previously described (26). Briefly, 10 μL of BALF supernatant was incubated for 30 min at 37°C, pH 5.0, protected from light, in the presence of 4-methylumbelliferyl-*N*,*N*′-diacetyl-b-d-chitobioside at 20 μg/mL (for chitobiosidase activity) in 100 μL final volume assay buffer. Reactions were stopped with sodium carbonate buffer and fluorescent signal was quantified using Synergy H1 micro-plate reader (Agilent), excitation 360 nm/emission 450 nm. Relative activity measurements were calculated after subtracting background values from wells containing substrate alone using a standard curve generated with serial dilutions of 4-methylumbelliferone. Chitinase from *Trichoderma viride* (Sigma) was used as a positive control and results from duplicate wells were averaged for all samples.

### In Vivo Chitin Staining

C57BL/6, *Chia^−^*^/−^, or SPAM mice were chronically exposed to *A. fumigatus* as described. Positive and negative controls for chitin staining included mice administered conidia that were pre-swollen for 6 h at 37°C for 2 h (positive) versus mice that were administered resting conidia for 2 h (negative) as we have previously described (17). Lungs were excised and placed in Z-fix for 24 h followed by an additional 24 h in fresh Z-fix. The fixed lungs were paraffin-embedded and processed by GNO Histology Consultants (New Orleans, LA). Lung tissue sections were deparaffinized with xylene, rinsed with 100% ethanol followed by 95% ethanol, and then finally with deionized water. Sections were stained using the Thermo Scientific Remel Calcofluor White Stain Kit (Fisher Scientific Cat. No. R40015) according to manufacturer’s instructions. Sections were imaged immediately after staining using a Nikon Eclipse T*i*2 fluorescent inverted microscope with NIS Elements imaging software (v.5.30.06).

### Statistics

Statistical analysis was performed using Prism (GraphPad). Pulmonary function metrics were analyzed using two-way ANOVA. Comparison between groups where data were normally distributed was analyzed with two-tailed Students *t* test, with a significance value accepted at *P* < 0.05.

## RESULTS

### Overexpression of Acidic Mammalian Chitinase Results in Worse Lung Function during Allergic Fungal Asthma

**Figure 1.**
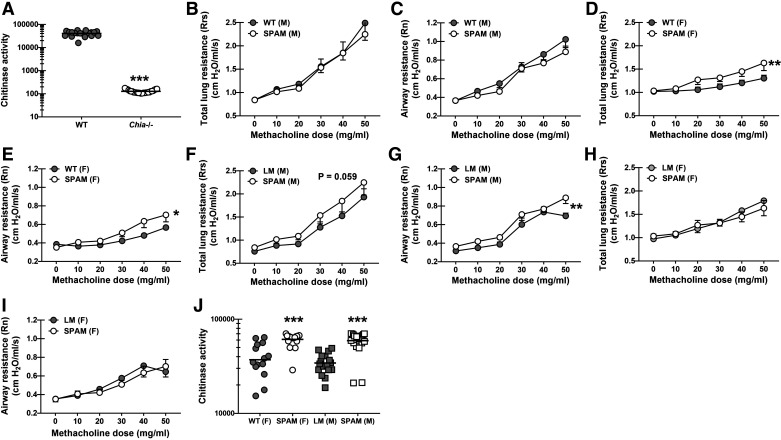
Overexpression of acidic mammalian chitinase results in worse lung function during allergic fungal asthma. Male and female C57BL/6 wild-type (WT) were subjected to the experimental allergic fungal asthma model as described in materials and methods. Twenty-four hours after the last challenge, chitinase activity was measured in clarified bronchoalveolar lavage (BAL) fluid. *A*: chitinase activity between female C57BL/6 WT and *Chia^−^*^/−^ mice. Figure *A* illustrates cumulative data from three independent studies (*n* = 5–9 mice/group, per study). Each dot represents a single mouse. The middle line represents the mean. ***P value of <0.0001 (two-tailed Student’s *t* test). Male and female C57BL/6 wild-type (WT) mice and mice overexpressing acidic mammalian chitinase (SPAM) and transgene negative littermates were subjected to the experimental allergic fungal asthma model as described in the materials and methods. Twenty-four hours after the last challenge, airway function was analyzed via mechanical ventilation using the flexiVent pulmonary function system. Total lung resistance between male WT and SPAM mice (*B*), airway resistance between male WT and SPAM mice (*C*), total lung resistance between female WT and SPAM mice (*D*), airway resistance between female WT and SPAM mice (*E*), total lung resistance between male littermates and SPAM mice (*F*), airway resistance between male littermates and SPAM mice (*G*), total lung resistance between female littermates and SPAM mice (*H*), and airway resistance between female littermates and SPAM mice (*I*). The figures (*B*–*I*) illustrate cumulative data from two to three independent studies (*n* = 4–6 mice/group, per study). Data are expressed as means ± SE. *,**P value of <0.05 and <0.01, respectively when comparing asthmatic WT or littermates to SPAM mice (two-way ANOVA). Male and female C57BL/6 wild-type (WT), mice overexpressing acidic mammalian chitinase (SPAM) and transgene negative littermates were subjected to the experimental allergic fungal asthma model as described in materials and methods. Twenty-four hours after the last challenge, chitinase activity were measured in clarified BAL fluid. *J*: chitinase activity between female WT and SPAM mice and male littermate and SPAM mice. The figure illustrates cumulative data from three independent studies (*n* = 4–6 mice/group, per study). Each dot represents a single mouse. The middle line represents the mean. ***P value of <0.0001 (two-tailed Student’s *t* test). [Image created with BioRender.com.]

Acidic mammalian chitinase (AMCase) is the predominant chitinase expressed in the lung (26) and is thus the primary factor dictating the level of chitin during exposure to chitin-bearing organisms. A previous study employing a relevant chitin-associated allergic asthma model by using house dust mite (HDM) and cockroach extracts surprisingly did not show a major role for AMCase in allergic asthma (27). In contrast, we previously reported that AMCase unexpectedly contributed to allergic fungal asthma severity as measured by lung function (17), suggesting that AMCase-mediated degradation of *A. fumigatus* chitin in vivo during allergic fungal asthma led to immune responses that increased sensitivity of the airways to bronchoconstrictors. Indeed, AMCase deficiency is associated with a significant reduction in lung chitinase activity during allergic fungal asthma (Fig. 1*A*). To further determine whether fungal chitin could affect lung physiologic responses during chronic fungal challenge, we subjected mice overexpressing AMCase (SPAM mice) (23, 25) to allergic fungal asthma. To compare the phenotype of SPAM mice with our previous report using AMCase-deficient (*Chia^−^*^/−^) mice (17), we first compared lung function with WT C57BL/6 mice. Results showed an unexpected sex difference, with no differences in total lung resistance (Fig. 1*B*) or airway resistance (Fig. 1*C*) between male BL/6 mice and male SPAM mice. In contrast, female SPAM mice had increased total lung resistance (Fig. 1*D*) and increased airway resistance (Fig. 1*E*) compared with female BL/6 mice. Regarding the latter, increased AHR in female SPAM mice correlated with increased IL-4, IL-5, IL-13, and IL-17A levels, although there were no changes in lung eosinophil or neutrophil levels (Supplemental Fig. S1; see https://doi.org.10.6084/m9.figshare.25901884). We next compared the lung function of SPAM mice with their littermate controls. A sex difference was again evident, however opposite than the comparison between WT C57BL/6 and SPAM mice. Male SPAM mice had a trend toward higher total lung resistance (Fig. 1*F*), but significantly higher airway resistance (Fig. 1*G*) compared with male littermate controls. There were no differences in total lung resistance (Fig. 1*H*) and airway resistance (Fig. 1*I*) between female littermates and female SPAM mice. As expected, elevated airway resistance in SPAM mice also correlated with an increase in lung chitinase activity (Fig. 1*J*). Note, there were no sex differences in lung chitinase activity (WT males in Fig. 1*A* vs. WT females in Fig. 1*J*, 41,018 ± 2,189, *n* = 20 vs. 37,104 ± 4,672, *n* = 15, *P* = 0.4168, as well as no differences between SPAM males and SPAM females in Fig. 1*J*). Thus, AMCase overexpression during allergic fungal asthma results in worse lung function that correlates with increased ability to break down chitin.

### Chitin-Bearing Organisms Are Detected in the Lung after Chronic *A. fumigatus* Exposure

**Figure 2.**
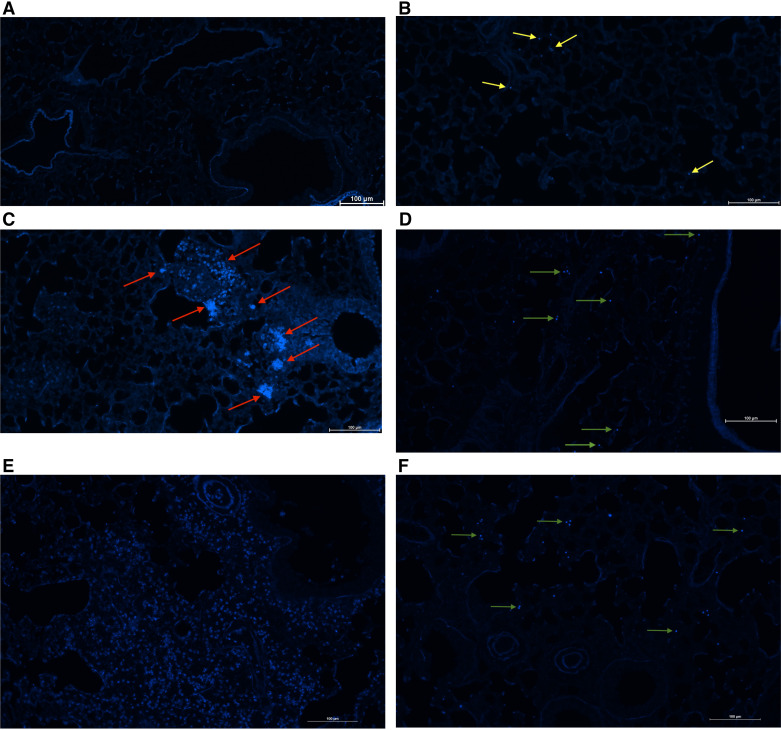
Chitin-bearing organisms are detected in the lung after chronic *Aspergillus fumigatus* exposure. C57BL/6 wild-type (WT), *Chia^−^*^/−^, and acidic mammalian chitinase overexpressing (SPAM) mice were intratracheally challenged with 1 × 10^7^ resting or swollen *A. fumigatus* conidia for 2 h or subjected to the experimental allergic fungal asthma model as in materials and methods. Lungs were collected, fixed, paraffin-embedded, and sectioned. Deparaffinized sections were stained using a Calcofluor White Stain Kit (Fisher Scientific Cat. No. R40015) according to manufacturer’s instructions. Sections were imaged using a Nikon Eclipse T*i*2 fluorescent inverted microscope with NIS Elements imaging software (v.5.30.06). Representative images (from 3 separate experiments with *n* = 3 mice/experiment) are shown for naïve mice (*A*), mice that received resting conidia (with yellow arrows marking chitin-positive conidia (*B*), mice that received swollen conidia (with red arrows marking chitin-positive swollen conidia) (*C*), mice subjected to allergic fungal asthma (with green arrows marking chitin-positive conidia) (*D*), SPAM mice subjected to allergic fungal asthma (*E*), and *Chia^−^*^/−^ mice subjected to allergic fungal asthma (*F*, with green arrows marking chitin-positive conidia). Original magnification ×20. Scale bar = 100 μm. [Image created with BioRender.com.]

Chitin is generally considered to be an immunopathogenic factor in allergic asthma. However, reports assessing its role in allergic asthma severity vary widely in the source of chitin used, exposure regiment, and outcome measures of severity. We have previously reported that chitin was detectable at low levels in resting *A. fumigatus* conidia in vitro, but was robustly observed in conidia that had swollen or germinated (17). As our chronic *A. fumigatus* exposure model uses repetitive challenge with resting conidia, we questioned whether fungal cell wall chitin was detectable in the lung during allergic fungal asthma. In our previous report, we found that calcofluor white was more sensitive than Alexa fluor 488-conjugated wheat germ agglutinin for detecting chitin in conidia over several time points (17). To validate our ability to detect chitin staining in a lung section, we first intratracheally challenged mice with *A. fumigatus* resting conidia or conidia that were pre-swollen for 6 h. Results showed a small amount of lung autofluorescence but no chitin-specific staining in naïve mice (Fig. 2*A*). Mice that received resting conidia had very little, albeit detectable chitin-positive conidia in the lung (Fig. 2*B*, yellow arrows). In contrast, mice that received pre-swollen conidia had very robust staining of chitin-positive conidia (Fig. 2*C*, red arrows), verifying our previous in vitro results (17) and confirming the ability of calcofluor white to stain *A. fumigatus* conidia in lung tissue. Note the increased size of *A. fumigatus* swollen conidia compared with the resting conidia in Fig. 2*B*. We next exposed mice to our chronic *A. fumigatus* exposure model to determine whether conidia displayed chitin in an allergic setting in vivo. Despite the relative rarity of conidia in the allergic lung (7) compared with invasive fungal pneumonia (28), these conidia nevertheless demonstrated staining with calcofluor white (Fig. 2*D*, green arrows). Note that conidia in the asthmatic lung appeared to be slightly larger than resting conidia in Fig. 2*B* yet smaller than swollen conidia in Fig. 2*C*. Conidia in asthmatic mice also appeared to stain more intensely than resting conidia in Fig. 2*B*. We also examined chitin staining in asthmatic SPAM mice, as these mice had elevated chitinase activity (Fig. 1*J*) and increased AHR (Fig. 1*E*). Intriguingly, we observed staining of chitin particles or digested chitin throughout the lung, suggesting that the heightened chitinase activity in SPAM mice was effective at liberating conidial chitin (Fig. 2*E*). Chitin staining of *A. fumigatus* conidia in asthmatic *Chia*^−/−^ mice appeared to be more frequent (Fig. 2*F*) than that observed in asthmatic WT mice (Fig. 2*D*), likely due to the lack of chitinase activity in the lungs of *Chia^−^*^/−^ mice. Thus, chitin is present in the lung during allergic fungal asthma.

### Chronic Exposure to Purified *A. fumigatus* Chitin Elicits Inflammatory Changes in the Lung

**Figure 3.**
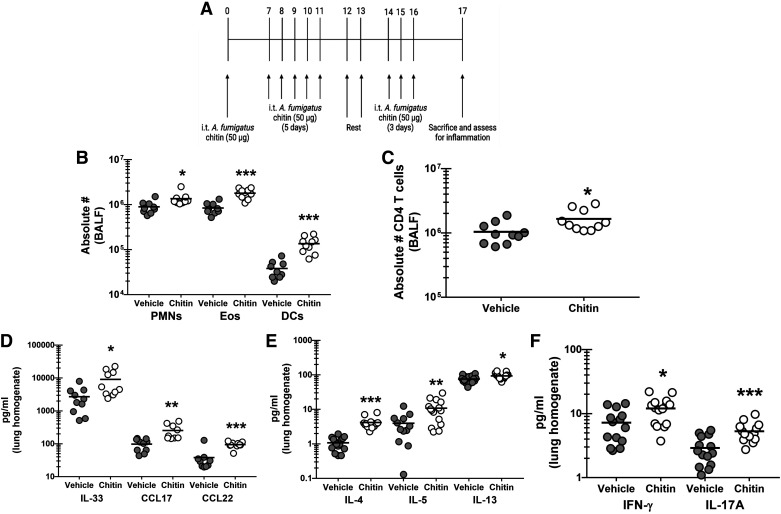
Chronic exposure to purified *Aspergillus fumigatus* chitin elicits inflammatory changes in the lung. C57BL/6 wild-type (WT) mice were exposed intratracheally to *A. fumigatus-*derived chitin as outlined in (*A*). Vehicle-treated mice received PBS at identical time points. Twenty-four hours after the last challenge, lung cells were isolated via bronchoalveolar lavage (BAL) were collected, homogenized, and clarified by centrifugation, enumerated, Fc-blocked, stained with a live/dead staining kit, and then stained using cell surface markers for neutrophils, eosinophils, and dendritic cells (*B*) or CD4 T cells (*C*) and quantified via flow cytometry. The figure illustrates cumulative data from two independent studies (*n* = 5 mice/group, per study). Each dot presents a single mouse. The middle line represents the mean. Data is expressed as absolute cell number in bronchoalveolar lavage fluid (BALF). *,**,***P value of <0.05, <0.01, and <0.0001, respectively (two-tailed Student’s *t* test). Twenty-four hours after last challenge, the left lungs were collected, homogenized and clarified by centrifugation. *D*: IL-33, CCL17, and CCL22 levels were quantified by ELISA. IL-4, IL-5, and IL-13 levels (*E*) and IFN-γ and IL-17A (*F*) were quantified by Milliplex. The figure illustrates cumulative data from three independent studies (*n* = 4 or 5 mice per group, per study). Each dot presents a single mouse. The middle line represents the mean. Data are expressed as pg/mL in lung homogenate. * and *** represent a *P* value of < 0.05 and < 0.0001, respectively (two-tailed Student’s *t* test). [Image created with BioRender.com.]

Data thus far have shown that chitin-positive *A. fumigatus* conidia are detected in the lung during allergic fungal asthma and the level of chitinase activity in the lungs directly correlates with AHR, suggesting that chitin breakdown drives AHR during allergic fungal asthma. To further define the specific impact of fungal chitin on allergic fungal asthma, we challenged WT BL/6 mice with chitin purified from *A. fumigatus* hyphae using the same exposure regimen that we use for our experimental allergic fungal asthma model using live *A. fumigatus* conidia (Fig. 3*A*). Twenty-four hours after the last chitin exposure, we observed ∼200%/2.1-fold increase in neutrophils, a ∼50%/1.5-fold increase in eosinophils and a ∼350%/3.5-fold increase in dendritic cells (Fig. 3*B*). Likewise, CD4+ T cells were increased ∼60%/1.6-fold (Fig. 3*C*). The increase in lung cellularity paralleled increases in the pro-type 2 factors IL-33, CCL17 and CCL22 (Fig. 3*D*), the type 2 cytokines IL-4, IL-5, and IL-13 (Fig. 3*E*) and increased induction of IFN-γ and IL-17A (Fig. 3*F*). Thus, chronic exposure to fungal-derived chitin provokes moderate inflammatory cell and inflammatory mediator changes in the lung.

### Chronic Exposure to Purified *A. fumigatus* Chitin Does Not Modulate Lung Physiology

**Figure 4.**
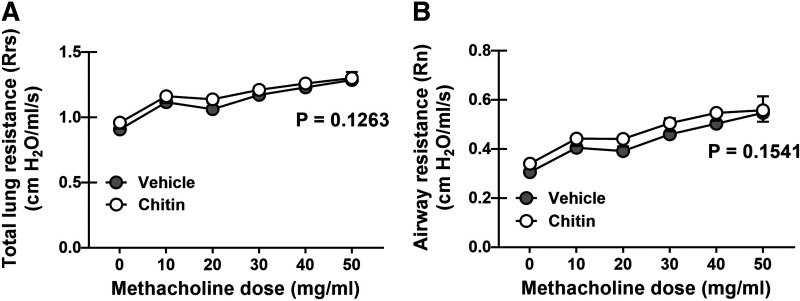
Chronic exposure to purified *Aspergillus fumigatus* chitin does not modulate lung physiology. C57BL/6 wild-type (WT) mice were exposed intratracheally to *A. fumigatus* derived chitin as in A. Twenty-four hours after the last challenge, airway function was analyzed via mechanical ventilation using the flexiVent pulmonary function system. Total lung resistance between vehicle (PBS) and chitin-exposed mice (*A*) and airway resistance between vehicle and chitin-exposed mice (*B*). The figure illustrates cumulative data from two independent studies (*n* = 5 mice/group, per study). Data are expressed as means ± SE (two-tailed Student’s *t* test). [Image created with BioRender.com.]

Chronic administration of purified fungal chitin increased lung cellularity, type 1, type 2, and type 17 responses, all of which we (7–10, 29) and others (30, 31) have shown can negatively affect lung function. Therefore, we next determined whether the inflammatory responses invoked by chronic fungal chitin exposure were sufficient to induce a change in lung function. Despite the proinflammatory and cellular changes that resulted from repeated chitin exposure, total lung resistance (Fig. 4*A*) and airway resistance (Fig. 4*B*) between vehicle and chitin-treated groups showed no differences. Thus, although having the ability to provoke a moderate inflammatory response, chronic exposure to fungal-derived chitin alone does not impact pulmonary function.

### Differences in Chitinase Activity Do Not Impact Lung Physiology during Chronic Exposure to Purified *A. fumigatus* Chitin

**Figure 5.**
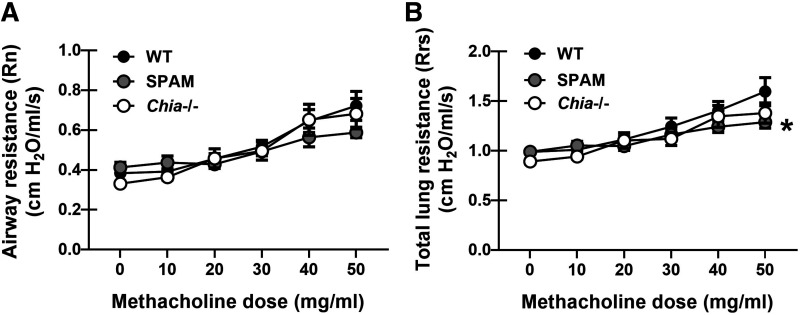
Differences in chitinase activity does not impact lung physiology during chronic exposure to purified *Aspergillus fumigatus* chitin. Male C57BL/6 wild-type (WT), mice overexpressing acidic mammalian chitinase (SPAM), and mice deficient in acidic mammalian chitinase (*Chia^−^*^/−^) were subjected to the chronic fungal chitin exposure as in Fig. 3*A*. Twenty-four hours after the last challenge, airway function was analyzed via mechanical ventilation using the flexiVent pulmonary function system. Airway resistance (*A*) and total lung resistance (*B*) between chitin-exposed WT vs. SPAM mice vs. *Chia^−^*^/−^ mice. The figures illustrate cumulative data from two to three independent studies (*n* = 4 or 5 mice/group, per study). Data are expressed as means ± SE. *P value of <0.05 when comparing asthmatic WT mice with SPAM mice (two-way ANOVA). [Image created with BioRender.com.]

Mice deficient in AMCase have little to no chitinase activity (Fig. 1) yet better lung function during allergic fungal asthma (17) whereas mice overexpressing AMCase have enhanced chitinase activity and worse lung function during allergic fungal asthma (Fig. 1). However, purified fungal chitin, while capable of provoking an immune response during chronic exposure in normal mice with homeostatic/sufficient chitinase activity, lacked the ability to influence lung function. As our data suggest that the ability to break down chitin dictates lung function during allergic fungal asthma, we questioned whether purified fungal chitin affected lung function in mice with differential chitinase activity. Despite showing that SPAM mice have heightened chitinase activity in the lung during allergic fungal asthma, chronic exposure of these mice to purified *A. fumigatus* chitin did not result in a change in airway resistance compared with vehicle-treated mice (Fig. 5*A*), although SPAM mice did show a small decrease in total lung resistance (Fig. 5*B*). Chronic exposure to purified *A. fumigatus* chitin did not induce any changes in total or airway resistance in *Chia^−^*^/−^ mice (Fig. 5, *A* and *B*). Thus, fungal chitin is unable to impact lung function, even in the presence of nearly absent (*Chia^−^*^/−^) or augmented (SPAM) chitinase activity.

## DISCUSSION

Although C/CLPs have been relatively well researched in asthma, there is still no agreement on whether they are promoters of disease or simply biomarkers of it (reviewed in Ref. 14). Indeed, we previously reported that the CLP YKL-40 was elevated in sputum from fungal-sensitized humans (18), which have more severe asthma (8), suggesting a pathogenic role for YKL-40. However, in an experimental model of allergic fungal asthma, we found that mice deficient in YKL-40 (BRP-39, *Chi3l1^−^*^/−^) actually have more severe asthma (18), suggesting a protective role for YKL-40/BRP-39. Studies have also implicated a role for AMCase in atopic asthma severity (32). In support of this, we have shown that AMCase contributes to severity in our experimental allergic fungal asthma model (17), although other models using relevant chitin-containing aeroallergens, i.e., house dust mite and cockroach, did not find a role for AMCase in severity (27, 33). Following up on the role of AMCase promoting allergic fungal asthma severity, we hypothesized that overexpression of AMCase in the lung would undoubtedly promote more severe fungal asthma. Indeed, our findings here support that, in that AHR was increased in SPAM mice when compared with the same WT control mice we previously used for AMCase-deficient mice (17) (females only) or with littermate controls (males only). This led to the initial hypothesis that AMCase-mediated degradation of chitin leads to worse lung function during allergic fungal asthma.

Despite significant research into the role of C/CLPs in asthma pathogenesis and severity, the role of chitin itself is much more obscure. Early studies challenging mice twice intranasally with a commercial chitin prep (source of chitin not provided) demonstrated increases in cells commonly associated with allergy, including eosinophils, basophils, and mast cells (25). A follow-up study by the same group examined the role of fungal-derived chitin in lung responses. Specifically, an alkali insoluble extract of *Aspergillus niger*, which was collected from dust isolated from the homes of human asthmatics, administered twice intranasally was shown to increase eosinophil recruitment to the lung one day after exposure (11). However, eosinophil recruitment was reduced when the extract was pretreated with either chitinase or β-glucanase, indicating that the extract did not contain pure fungal chitin and that fungal β-glucans were equally capable of recruiting eosinophils to the lung. This study also performed a more chronic exposure with the *A. niger* fungal extract (3 doses/wk for 3 wk) and demonstrated an increase in total lung resistance. However, it was curious to note that there was no difference in lung function between mice with lower (4get mice) versus higher (4get x SPAM mice) chitinase activity, again likely owing to the contaminating β-glucans in the *A. niger* fungal extract driving changes in total lung resistance in both strains (11). A similar study used intratracheally challenged mice with shellfish-derived chitin in the presence or absence of a commercial *Aspergillus* extract using a more chronic exposure regiment, challenging mice every 4 days for five times. This resulted in changes in lung function (without administration of airway provocation agents such as methacholine, which is a hallmark of defining asthma as a diagnosis) and eosinophil recruitment. Eosinophil recruitment here required administration of both chitin and the extract, as neither alone had an effect, and was dependent on complement C3 and C3R (21). Acute challenge with chitin-coated beads (administered twice) has been shown to induce IL-23, IL-33, and thymic stromal lymphopoietin (TSLP) production coupled with eosinophil and ILC2 recruitment (23). Likewise, shellfish-derived chitin administered once, twice, or three times can drive IL-33-dependent eosinophilia (19). Shellfish-derived chitin coadministered with ovalbumin (OVA) has been reported to have an adjuvant-type effect driving IL-33 production and type 2 responses (20). However, none of these latter studies assessed the impact on these various chitin exposure regimens on lung function (19, 20, 23). Studies also argue against a negative role for chitin in asthma, as an early study indicated that chitin microparticles derived from shrimp administered prior to OVA sensitization were protective against asthma (34). Similar results were observed with chitosan (deacetylated chitin) and house dust mite (35).

Our initial hypothesis was that mice deficient in AMCase subjected to our experimental allergic fungal asthma model would have higher chitin levels in the lung due to the lack of chitinase activity, which we expected would lead to worse lung function. As stated earlier, we unexpectedly discovered that AMCase-deficient mice had less severe asthma (17) in the presence of lower chitinase activity. Likewise, AMCase overexpressing mice had worse lung function in the presence of higher chitinase activity. Therefore, the direct connection between chitinase activity and lung function suggested that chitin played a role in allergic fungal asthma responsiveness. Coupled with a lack of published data on the effects of chronic purified fungal chitin exposure, we questioned whether fungal chitin was a relevant, aeroallergen in terms of eliciting inflammation and driving a change in lung physiological responses. To specifically address this, we used chitin derived from a fungal species that has a known association with asthma (*A. fumigatus*) and administered it chronically (9 times over 16 days) to mice and analyzed changes in lung cellularity, proinflammatory, type 1, type 2, type 17 responses as well as pulmonary function. Similar to previous reports, whether the chitin was shellfish-derived or the administration was acute or chronic, fungal chitin induced an increase, albeit small, in eosinophils. Likewise, fungal chitin induces type 2-associated mediators, such as IL-33 and type 2 cytokines, as well as IFN-γ and IL-17A, the latter of which was also associated with increased neutrophil levels in the lung. Therefore, chronic administration of fungal-derived chitin appeared “immunologically capable” of driving a change in lung function. However, total lung resistance and airway resistance/AHR were not different between vehicle and chitin-treated mice. This does not necessarily mean that fungal chitin plays no role in asthmatic lung physiologic responses; our data suggest however that it is unable to do so alone in a chronic exposure regimen. We posit here that additional components of the fungal cell well, such as β-glucans, must also be liberated in conjunction with chitin to pass a threshold of inflammation required to induce AHR during allergic fungal asthma. Data supporting this come from our previous report indicating that Dectin-1-mediated recognition of β-glucan drives significant inflammatory responses and AHR (7). Moreover, we show that SPAM mice have increased AHR in the presence of diffuse staining of chitin particles throughout the lung, suggesting that increased chitinase activity in SPAM mice liberated chitin, and potentially other fungal pathogen-associated molecular patterns (PAMPs) (i.e., β-glucan), from the conidial cell wall. In addition, as chitin is a major component of the insect exoskeleton, our findings may have relevance to known asthma-associated allergens such as cockroach and house dust mite, in that inflammation-driven responses to AHR during chronic exposure to cockroach or house dust mite extracts may be provoked by both chitin and other allergens in these preparations [e.g., the Der p1 cysteine proteinase from house dust mite (36)]. Our results are in disagreement with a previous report showing that shellfish-derived chitin administered daily for 3 days induced AHR (22). However, this report used Rrs (also known as R) to define AHR. Rrs is a measure of total lung resistance, which measures resistance in both the conducting/proximal and distal airways. In contrast, Rn (also known as Raw) is termed Newtonian resistance or resistance of the large conducting airways, and is considered a more accurate measure of AHR (37), particularly during experimental allergic asthma (38). Moreover, this report only found differences at the highest methacholine dose (48 mg/mL, which is similar to our highest dose of 50 mg/mL) and used a one-way ANOVA to make statistical comparisons within this highest dose instead of using a two-way ANOVA, which makes statistical comparisons across all doses [as we have extensively reported (8–10, 17, 18)]. This report also showed other conflicting data that chitin alone did not induce an increase in Rrs over untreated mice at the 48 mg/mL methacholine dose, whereas OVA induced a robust, steroid-regulated increase in Rrs that was higher, but not significant, when chitin was coadministered with OVA during steroid treatment (22).

Some reports have asserted that the size of chitin can either promote or restrict the host immune response, thereby dictating disease pathogenesis and severity. For example, one report separated shellfish-derived chitin by size in small (less than 40 μm; termed cleaved chitin), intermediate (40–70 μm), and large (70–100 μm, termed uncleaved chitin) preparations and examined inflammatory responses and induction of AMCase (39). This report demonstrated that intranasal challenge with large chitin induced more *Chia* expression and recruited more eosinophils to the lung than small chitin. Large chitin also induced IL-33 whereas small chitin did not. However, small chitin was better at inducing IL-1β than large chitin (39). A similar report generated small chitin (<40 μm), intermediate chitin (40–70 μm), and big chitin (>70 μm) and demonstrated that small chitin promoted TNF-α induction in the lung whereas intermediate chitin predominantly drove IL-10 induction (40). Our *A. fumigatus* chitin preparation would fall into the small chitin size, with more than 70% of this preparation consisting of chitin particles less than 0.5 μm (24). These much smaller particles are still immunoreactive when administered to the lung and are likely broken down further to oligomeric chitin. Indeed, oligomeric chitin (derived from crab shells) of at least six *N*‐acetyl d‐glucosamine units (10–15 U/∼5 nm was optimal) is sufficient for inducing IL-6 and TNF-α production by human and murine macrophages and recruiting neutrophils to the lung after intranasal challenge (41).

In summary, *1*) we used chitin sourced from a prominent etiologic agent of allergic asthma (the mold *A. fumigatus*), *2*) the fungal chitin preparation was primarily comprised of very small particles (<0.5 μm), *3*) we administered fungal chitin chronically over 16 days, and *4*) we examined the effects of chronic fungal chitin exposure on immunologic and physiologic (lung function) responses. Collectively, we found that chronic exposure to chitin alone was not sufficient for inducing AHR nor were there differences in AHR when lung chitinase levels were nearly absent or significantly elevated. Our findings therefore provide insight into an unexpected noncontributory role of chitin in allergic fungal asthma severity.

## DATA AVAILABILITY

Data will be made available upon reasonable request.

## SUPPLEMENTAL MATERIAL 

10.6084/m9.figshare.25901884 Supplemental Fig. S1: https://doi.org.10.6084/m9.figshare.25901884.

## GRANTS

This work was supported by National Institutes of Health Grants HL122426 and HL136211 (both to C.S.).

## DISCLOSURES

No conflicts of interest, financial or otherwise, are declared by the authors.

## AUTHOR CONTRIBUTIONS

D.A.E., V.A., and C.S. conceived and designed research; D.A.E., M.J., H.M.E.W., S.C., and M.M. performed experiments; D.A.E., M.J., and C.S. analyzed data; D.A.E. and C.S. interpreted results of experiments; D.A.E. and C.S. prepared figures; D.A.E. and C.S. drafted manuscript; D.A.E. and C.S. edited and revised manuscript; D.A.E. and C.S. approved final version of manuscript.
